# Expression of the cholesterol transporter SR-B1 in melanoma cells facilitates inflammatory signaling leading to reduced cholesterol synthesis

**DOI:** 10.1016/j.neo.2025.101154

**Published:** 2025-03-21

**Authors:** Oliver Eckel, Madalina A. Mirea, Anna Gschwendtner, Martina Pistek, Katharina Kinslechner, Clemens Röhrl, Herbert Stangl, Markus Hengstschläger, Mario Mikula

**Affiliations:** a Institute of Medical Genetics, Center for Pathobiochemistry and Genetics, Medical University of Vienna, Währinger Strasse 10, 1090, Vienna, Austria; b University of Applied Sciences Upper Austria, Faculty of Engineering, Stelzhamerstraße 23, 4600, Wels, Austria; c Institute of Medical Chemistry, Center for Pathobiochemistry and Genetics, Medical University of Vienna, Währinger Strasse 10, 1090, Vienna, Austria

**Keywords:** HDL receptor, Cholesterol, S1P, HIF1A, Inflammation, BLT-1, Statin, Tumor organoid, Skin-organoid

## Abstract

Scavenger receptor class B type 1 (SR-B1) is a cholesterol transporter, abundantly expressed in human melanoma, yet its precise role for melanoma progression is not fully understood. This study investigates the involvement of SR-B1 in cholesterol homeostasis of tumor cells and its implications for potential therapy. We found that SR-B1 depletion in melanoma cells does not alter total cholesterol levels, but induces cholesterol biosynthesis. This effect was characterized by an increased expression of HMG-CoA reductase (HMGCR), a rate limiting enzyme of cholesterol biosynthesis. Notably, further analyses indicated that this regulation occurs at the post-translational level, mediated via the hypoxia-inducible factor (HIF) signaling pathway. Importantly, we identified SR-B1 as a transporter of the lipid hormone sphingosine-1-phosphate (S1P) and we found that S1P exposure leads to HIF1A up-regulation. Finally, we used a pluripotent stem cell-derived skin organoid model to show that targeting SR-B1 in combination with targeted melanoma therapy can lead to increased apoptosis and suppressed proliferation of transplanted tumor cells. Our study shows that functional SR-B1 is linked to inflammatory signaling, which reduces cholesterol synthesis, while enabling melanoma cell survival during chemotherapy treatment.

## Introduction

Malignant melanoma is a highly aggressive form of skin cancer with a rising incidence and significant mortality worldwide [1]. Despite advances in therapeutic approaches, melanoma remains a challenging cancer to treat, particularly in advanced stages. One of the emerging hallmarks of melanoma malignancy is its ability to modulate lipid metabolism, specifically cholesterol homeostasis, which plays a critical role in tumor progression and metastasis [2]. Elevated cholesterol levels have been associated with increased cellular proliferation, invasion, and resistance to apoptosis in melanoma cells, making cholesterol metabolism a key factor for understanding melanoma biology and developing potential therapeutic strategies [3].

Scavenger receptor class B type 1 (SR-B1) is a high-affinity receptor that mediates selective uptake of cholesterol esters from high-density lipoproteins (HDL) [4]. SR-B1 is highly expressed in organs involved in cholesterol metabolism like liver and adrenal glands [[5], [6], [7]]. Recently, SR-B1 has also been found to be associated with tumor formation in prostate, skin and breast cancer [[8], [9], [10]]. In breast and nasopharyngeal carcinoma the receptor is functionally implicated in the accumulation of cholesterol and it not only supports the structural integrity of cell membranes and the formation of cholesterol rich lipid rafts, but also contributes to signaling pathways that promote cell survival and proliferation [8,11]. Given the critical role of SR-B1 in cholesterol transfer, SR-B1 represents a potential therapeutic target for melanoma therapy, since its blockade could disrupt the cholesterol balance and hinder tumor growth.

Inflammation is a process frequently associated with melanoma development [12]. In later stages it hallmarks cancer cell stress and enables migration and invasion of tumor cells into the surrounding tissue [13]. Main drivers of inflammation include HIF1A activation, as well as S1P signaling. Interestingly, HIF1A has been shown to influence cholesterol balance, as it controls HMG-CoA reductase (HMGCR) protein amounts post-translationally, by ubiquitination and ER-associated degradation [14,15]. Hence, HIF1A activity represents a major factor for the control of cholesterol synthesis in cells and tissues. It is important to study the implications of SR-B1 and its associated HIF1A activity. Importantly, the lipid hormone sphingosine-1-phosphate (S1P) has been identified as a non-hypoxic activator of HIF1A in endothelial cells and also carcinoma cells [16,17]. As a result, S1P could represent a potential link between SR-B1 expression and HIF1A activation in melanoma.

The present study investigated the role of SR-B1 on cholesterol synthesis by investigating specifically HIF1A and S1P signaling. Furthermore, we analyzed the effects of SR-B1 inhibitors, in addition to cholesterol synthesis inhibitors, on melanoma cell survival and proliferation during chemotherapy treatment by employing a novel organoid-based model system.

## Results

### SR-B1 is highly expressed in malignant melanoma and regulates cholesterol synthesis

**Fig. 1:**
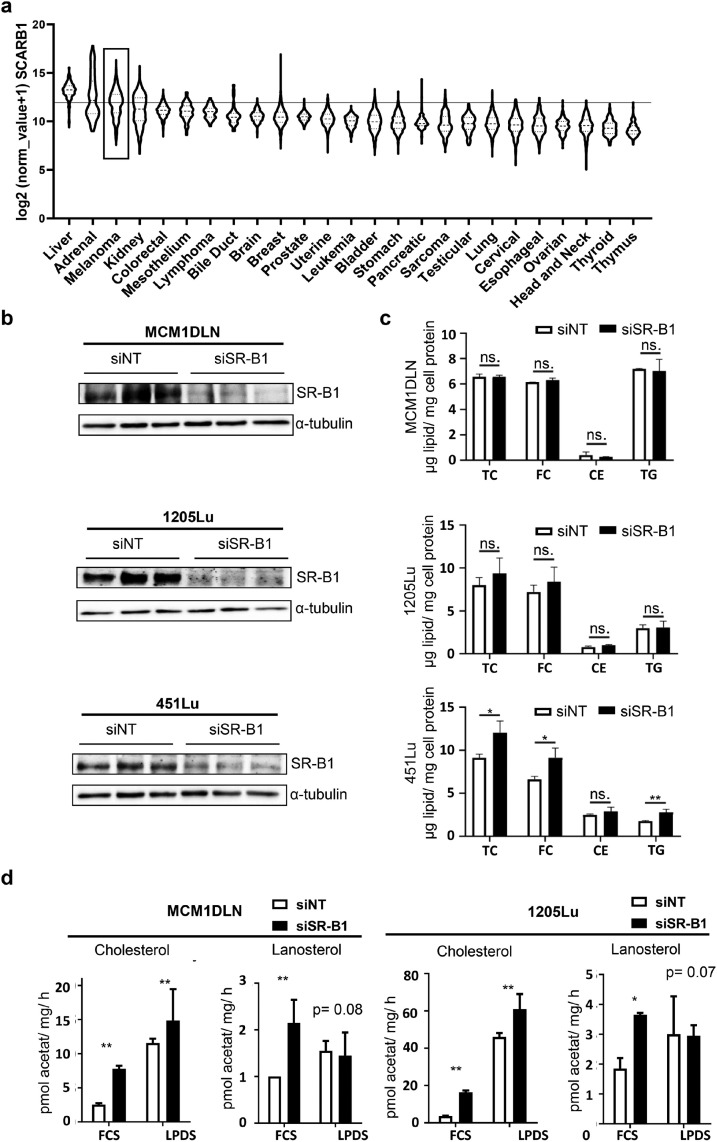
Expression of SR-B1 hallmarks malignant melanoma development and its loss induces cholesterol synthesis. **(a)** SR-B1 mRNA expression in different cancer types depicted from the TCGA PANCAN dataset. **(b)** Three independent, late stage, melanoma cell lines were cultured and 48 hours after control or siSR-B1 treatment proteins were harvested. Knockdown efficiency for SR-B1, shown by western blotting. Alpha-tubulin was used as loading control. **(c)** From the same experimental setup lipids were extracted, measured by gas chromatography and normalized to total cellular protein. (**d)** Cholesterol synthesis was measured in indicated SR-B1 knockout and control cells by trace-labeling cells with radioactive acetate and measuring metabolic conversion to cholesterol as well as lanosterol. TC= total cholesterol, FC= free cholesterol, CE= cholesterol ester, TG= triglycerides. FCS= normal serum condition, LPDS= lipoprotein-deficient serum. * = P < 0.05, ** = P < 0.01.

To highlight the importance of SR-B1 in malignant melanoma, we analyzed SR-B1 expression across major human tumors using data from the TCGA consortium. As expected, liver and adrenal tumors exhibited the highest SR-B1 expression (Fig. 1a). Notably, melanoma samples ranked third in SR-B1 expression among all tumor types. Independent database analysis confirmed significantly higher SR-B1 levels in melanoma tissues compared to normal skin samples (Supplementary Fig. S1a). Furthermore, melanoma samples with elevated SR-B1 expression demonstrated increased skin invasion (Supplementary Fig. S1b).

Given that SR-B1 functions as a cholesterol transporter, we assessed lipid levels and composition following SR-B1 knockdown using siRNA in three melanoma cell lines (Fig. 1b). Lipid extraction and quantification revealed no reduction in free cholesterol, cholesterol esters, or triglycerides (Fig. 1c). Surprisingly, one cell line even exhibited a significant increase in cholesterol. To further examine cholesterol homeostasis, we measured cholesterol synthesis in cells grown with and without lipoproteins (Fig. 1d). SR-B1 depletion by siRNA significantly increased cholesterol synthesis in both conditions, while lipid starvation generally enhanced cholesterol production. Steady state levels of lanosterol, a cholesterol precursor, showed a significant increase after SR-B1 knockdown, but only in the presence of lipids.

### HMGCR mRNA expression remains unchanged, but protein is upregulated after SR-B1 knockdown

**Fig. 2:**
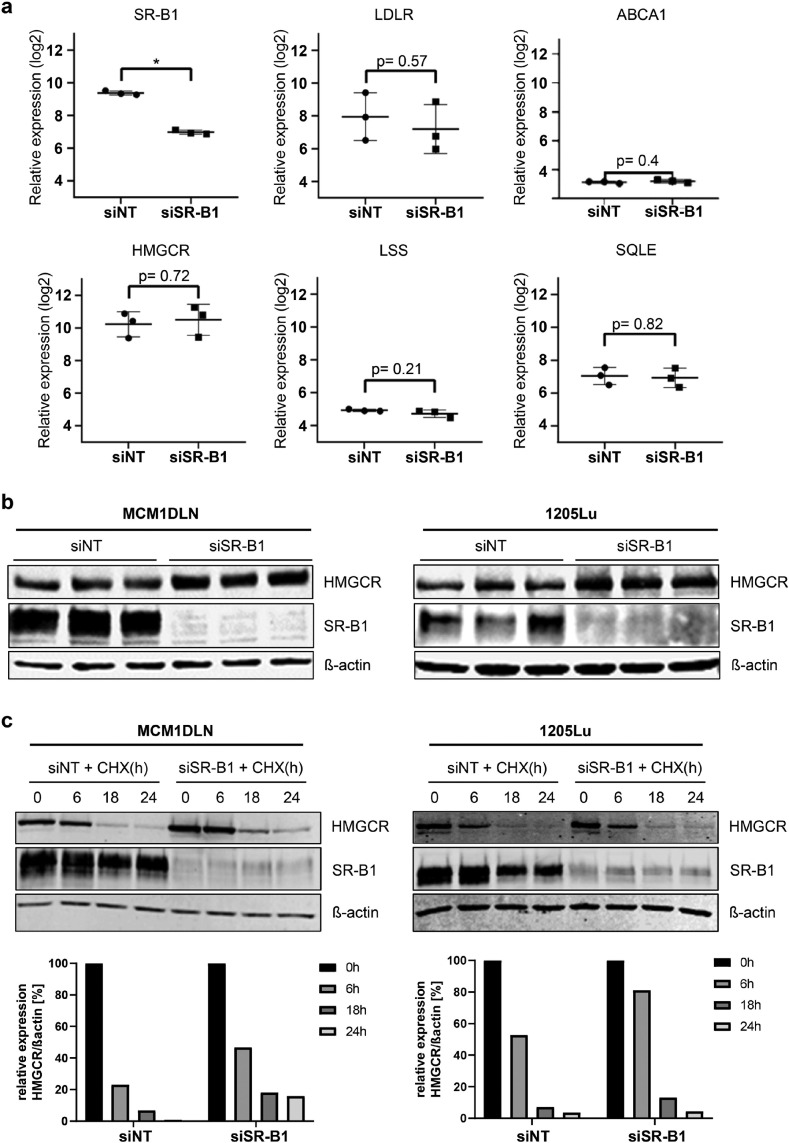
SR-B1 regulates HMGCR protein amounts post-translationally. **(a)** Regulation of SR-BI and genes involved in cholesterol uptake (LDLR), secretion (ABCA1) and synthesis (HMGCR, LSS, SQLE) 48 hours after siRNA control or siRNA SR-B1 treatment, as measured by expression arrays. * = P < 0.05. **(b)** Protein amounts of HMGCR, as well as SR-B1, were measured in triplicate 48 hours subsequent to control or siRNA SR-B1 treatment by western blot. Beta-actin was used as loading control. **(c)** Analog to experiment shown in (b), but with additional cycloheximide treatment to block de-novo protein translation. Normalized protein quantification displayed in bar chart. CHX= cycloheximide.

To investigate the observed upregulation of cholesterol synthesis, we quantified mRNA levels of key cholesterol homeostasis genes. While SR-B1 mRNA was effectively downregulated, no changes were observed in the expression of transporters low density lipoprotein receptor (LDLR), ATP-binding cassette transporter (ABCA1) or enzymes like HMG-CoA Reductase, Lanosterol Synthase (LSS) and Squalene Epoxidase (SQLE) (Fig. 2a). However, HMGCR, the rate-limiting enzyme for cholesterol synthesis, was upregulated at the protein level following SR-B1 knockdown (Fig. 2b and Supplementary Fig. 2). To rule out the contribution of *de novo* HMGCR protein synthesis, we repeated the experiment while cells were treated with cycloheximide, a protein synthesis inhibitor (Fig. 2c). Quantification of normalized HMGCR protein showed delayed degradation in SR-B1 devoid cells.

### Loss of SR-B1 reduces HIF1A levels

**Fig. 3:**
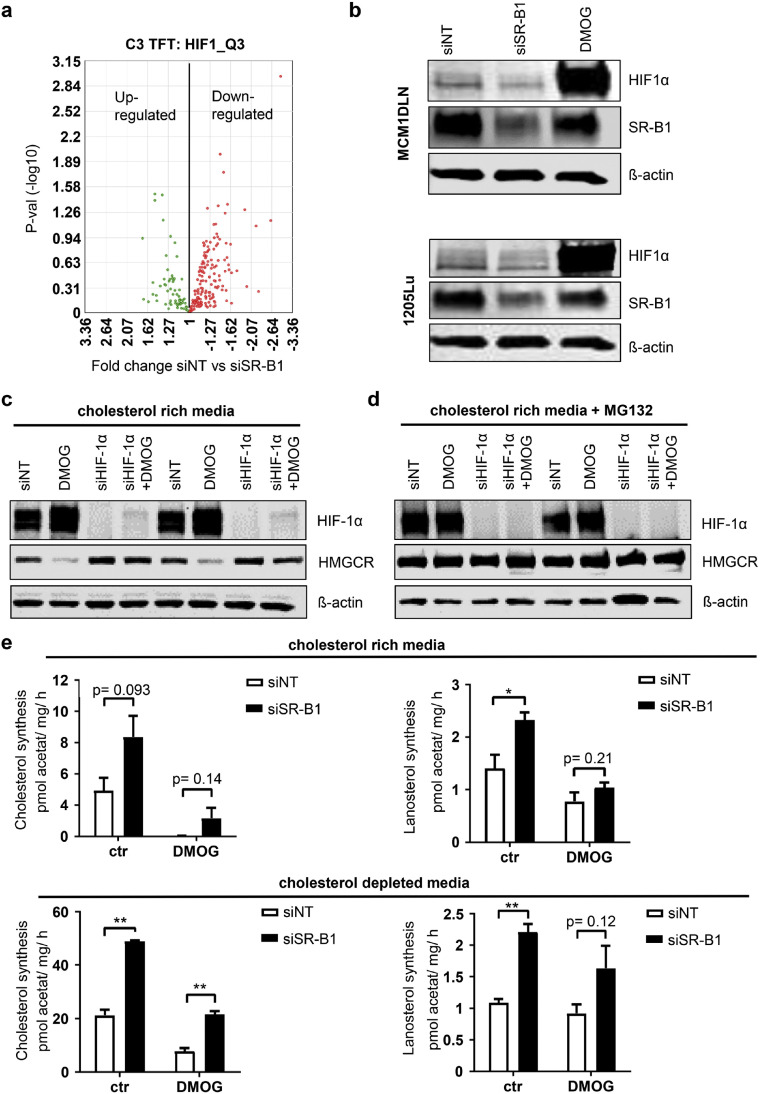
SR-B1 regulates HIF1A amounts and HIF1A degrades HMGCR via the proteasome. **(a)** Volcano plot including three different melanoma cell lines, 48 hours after siRNA SR-B1 transfection, compared to control, showed down-regulation of HIF1 target genes included in the GSEA C3 TFT: HIF_Q3 gene set. **(b)** Effect of siRNA targeting of SR-B1 on HIF1A levels shown by Western blot. DMOG treatment lasted for 6 hours and beta-actin was used as a loading control. **(c)** To show the impact of HIF1A on HMGCR, cells were treated with siRNA control, DMOG, siRNA HIF or in combination and used for western blotting. Beta-actin was used as loading control. **(d)** Setup shown in (c) was repeated, but with additional incubation of the proteasome inhibitor MG132. **(e)** Effect of HIF induction on cholesterol synthesis was tested in cholesterol rich growth conditions. 48 hours prior cells were either siRNA control or siRNA SR-B1 treated, 6 hours prior DMOG was applied, then labeled acetate was added to the media. Subsequently lipid was extracted and labeled cholesterol as well as lanosterol was measured by thin chromatography, normalized to total protein amounts and divided by incubation time (n=3). **(f)** Same experiment as in (e) was performed, but cells were cultured in cholesterol depleted medium, hence higher amounts of synthesis were measured. (n = 3). * = P < 0.05, ** = P < 0.01.

HIF1A activity has been implicated in regulating HMGCR degradation [14]. We analyzed HIF1A effects depending on the presence of SR-B1. We used the GSEA annotated HIF1A target genes and found that 66% of probe sets were downregulated after SR-B1 knockdown (Fig. 3a). Western blot analysis confirmed reduced HIF1A levels. Treatment with DMOG, an inhibitor of HIF1 protein degradation, served as a positive control (Fig. 3b).

To examine the effect of HIF1A on HMGCR amounts, cells were treated with DMOG, siRNA targeting HIF1A, or both. Induction of HIF1A drastically reduced HMGCR levels, while HIF1A knockdown rescued this effect (Fig. 3c). Additionally, treatment with MG132, a proteasome inhibitor, indicated that ubiquitination-dependent degradation mediated HMGCR regulation (Fig. 3d). Cholesterol synthesis assays performed with DMOG treatment revealed reduced cholesterol production, underpinning the impact of HIF1A on cholesterol synthesis (Fig. 3e).

### SR-B1 drives S1P signaling

**Fig. 4:**
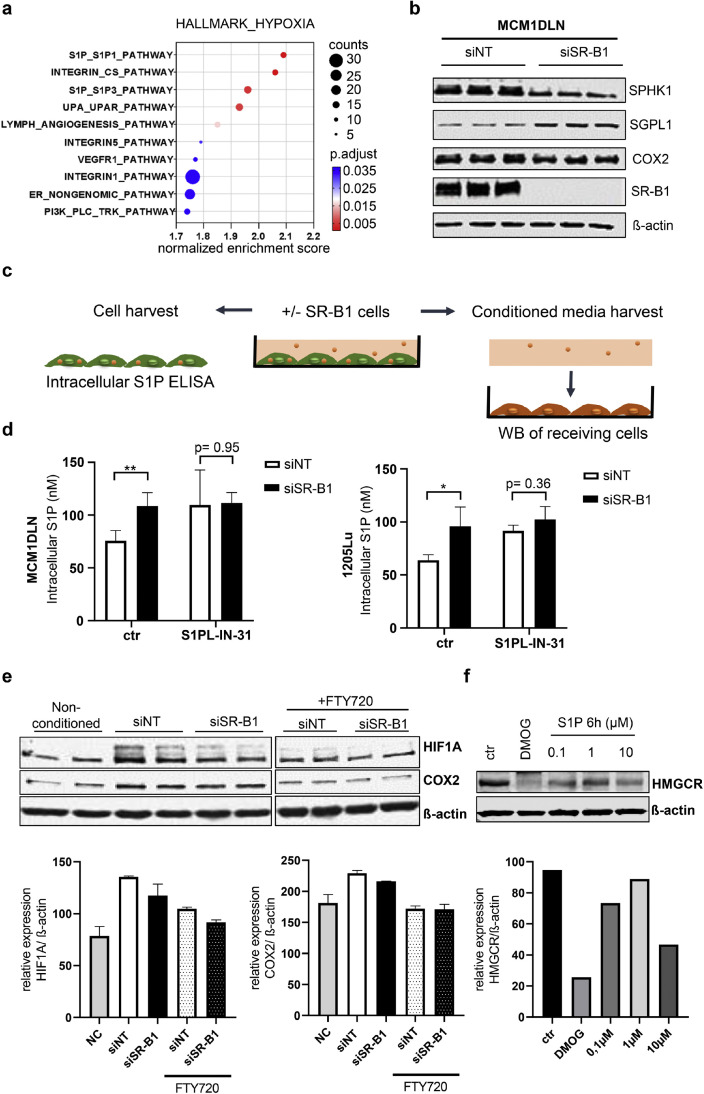
SR-B1 drives S1P signaling, which up-regulates HIF1A and down-regulates HMGCR. **(a)** Pathway enrichment in control versus 48 hours siRNA SR-B1 treatment was analyzed by plotting GSEA C2:CP:PID genesets for their respective normalized enrichment score, gene count and adjusted p-value. **(b)** Western blotting for S1P kinase (SPHK1), S1P lyase (SGPL1) and the S1P induced gene COX2, in siRNA SRB1 treated and control cells. SR-B1 was used to confirm the knockdown and beta-actin was used as a loading control. **(c)** Schematic for isolation of cellular protein for ELISA and conditioned medium for activation assays as shown in subsequent experiments. **(d)** 48 hours’ prior to harvesting, cells were either control or siRNA SRB1 transfected, plus treatment with the S1P lyase inhibitor S1PL-IN-31 served as a control. Total protein was used for S1P specific ELISA measurement. (n = 4 / cell line and treatment). * = P < 0.05, ** = P < 0.01. **(e)** 1205Lu melanoma cells were incubated with either normal media, conditioned media from control treated cells or conditioned media from siRNA SRB1 treated cells. The latter two were also mixed with S1P receptor inhibitor fingolimod (FTY720). Recipient cells (also 1205Lu) were then harvested for western blotting with detection of HIF1A and COX2. Beta-actin was used as loading control. Normalized protein quantification displayed in barchart. **(f)** Western blotting was performed to measure the amounts of HMGCR after exposure to S1P with the indicated concentrations. DMOG was used as a positive control. Normalized protein quantification displayed in bar chart.

To link SR-B1 with HIF1A regulation, we performed a genomic screen that highlighted enrichment of the S1P pathway in control cells compared to SR-B1 knockdown cells (Fig. 4a). Expression analysis of S1P pathway components, including Sphingosine Kinase 1 (SPHK1), which enhances S1P levels and S1P Lyase (SGPL), which degrades S1P, was performed. Also, the downstream target and inflammation mediator COX2 was analyzed. Results confirmed that SR-BI is necessary for enhanced S1P signaling (Fig. 4b).

Melanoma cells are known to gain self-sufficiency by establishing tumor promoting feedback loops [[18], [19], [20]]. In order to assess how SR-B1 regulates autocrine S1P signaling an experimental plan was conceived in order to investigate intracellular as well as extracellular-derived samples (Fig.4c). Cytoplasmic S1P levels were quantified using specific ELISAs, which revealed a significant increase following SR-B1 knockdown (Fig. 4d). This increase also matched levels, which were observed with S1P lyase inhibitor treatment, suggesting that SR-B1 facilitates S1P export. Furthermore, media, conditioned by culturing either control or si SR-B1 treated cells, was collected and used to stimulate quiescent melanoma cells. This experiment suggests that media from SR-B1 knockdown cells contained less S1P compared to control media and hence was less effective at inducing HIF1A and COX2 (Fig. 4e), which is in line with the finding that SR-B1 knockdown leads to a retention of S1P within cells. The addition of fingolimod (FTY720), a SIP receptor 1 and 3 inhibitor, showed that the effect of conditioned media was mediated by S1P receptor activation. Additionally, treatment with 10 μM of S1P led to reduced HMGCR levels in 1205Lu and MCM1DLN cells (Fig. 4f and Supplementary Fig. 3).

### Combined pharmacologic treatment of tumor organoids

**Fig. 5:**
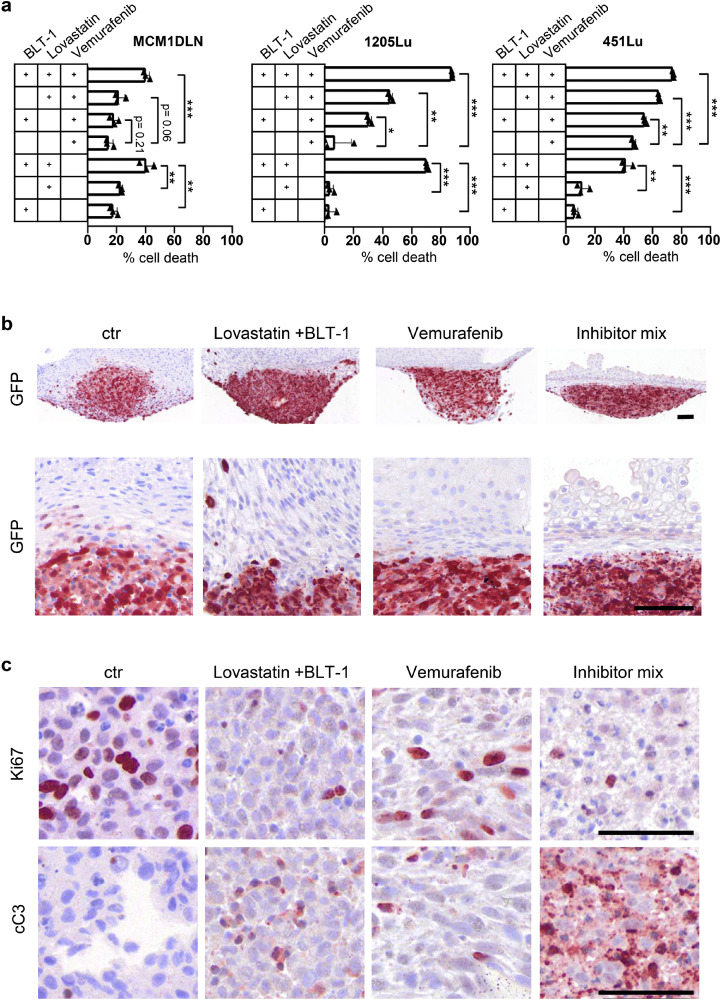
Combined pharmacologic treatment of tumor organoids reveals synergistic effects. **(a)** Indicated independent melanoma lines were treated with a selective cholesterol transport inhibitor (100nM BLT-1), Lovastatin (1000nM), Vemurafenib (500nM), as well as with combinations thereof. Cytotoxicity was assessed 24 hours later by quantifying the ability of cells to reduce resazurin. * = P < 0.05, ** = P < 0.01, *** = P < 0.001. **(b)** Immunohistochemistry on sections of human skin organoids 14 days after tumor spheroids were grafted and incubated with identical concentrations of inhibitors as shown in (a). Immuno-histochemical staining for GFP was used to localize tumor tissue. Magnifications show invasion of tumor cells into dermal tissue. **(c)** Consecutive sections within the tumor area were stained for Ki67, a marker for cycling cells, and active caspase 3 (cC3), a marker for apoptosis, after application of the indicated treatments. Inhibitor mix= Lovastatin+ BLT-1+ Vemurafenib. Scale bar = 50 µm.

In order to study the effect of SR-B1 function, on melanoma cell growth and survival, it is crucial to make use of available pharmacologic inhibitors. BLT-1 (Block Lipid Transport 1) is a specific SR-B1 inhibitor, which was developed to selectively block HDL mediated cholesterol uptake. The capability to reduce inflammatory marker expression was tested for SR-B1 knockdown as well as for BLT-1 treatment and both conditions showed a similar effectiveness (Supplementary Fig.4). Importantly S1P triggers the NFkB pathway, hence only genes included in this hallmark geneset, for example: COX2 and SPHK1, were used to demonstrate downregulation. Furthermore, the impact of BLT-1 in combination with the cholesterol biosynthesis inhibitor lovastatin and the targeted chemotherapy compound Vemurafenib was tested for its influence on the viability of melanoma cells (Fig. 5a). Importantly, the combination of the cholesterol transport inhibitor with the cholesterol synthesis inhibitor yielded a highly significant upregulation of cell death and the combination of all three inhibitors showed the strongest response. In order to establish a more *in vivo* like environment we generated human pluripotent stem cells-derived skin organoids and used 40 days old organoids as hosts for GFP-labelled melanoma grafts. Tumor organoids were continuously treated with identical inhibitor concentrations as used in Fig.5a and tumor tissue from the invasion front was harvested 14 days later. The use of statin, as expected, led to increased protein amounts of SR-B1 and HMGCR in the tumor tissue (Supplementary Fig. 5). Investigation of sections showing the tumor-host border revealed that control as well as statin plus BLT-1 treatment still showed pronounced tumor cell invasion (Fig. 5b), while targeting BRAFV600E by using Vemurafenib prevented penetration of tumor cells into the organoid. Interestingly, combinatory use of all inhibitors showed the strongest effect on tumor cells, while the rest of the tissue stayed morphologically unaffected. On the other hand, statin plus BLT-1 reduced cell proliferation, as evidenced by Ki67 antibody staining, and increased cellular apoptosis, as shown by active caspase staining (Fig. 5c). Importantly, the combination of all inhibitors showed the strongest effect. This indicates that blocking of SR-B1 function alone is not sufficient to decrease tumor growth in our model, but that combination with statin drives tumor cell apoptosis. Invasion seemed unaffected, but could be reduced by the application of Vemurafenib.

## Discussion

In this study, we showed that loss of SR-B1 reduced HIF1A levels and concomitantly increased cholesterol synthesis. Additionally, we showed that SR-B1 regulated HMGCR stability and that also its transporting activity was necessary for the survival of melanoma cells during targeted therapy.

The amount of cholesterol within cells is tightly regulated and oversupply as well as undersupply is immediately counterbalanced due to strict regulation pathways [21]. So, the question arises: why does the loss of SR-B1 in melanoma cells not alter the intracellular cholesterol amounts? This can be explained mainly by the well acknowledged upregulation of cholesterol synthesis, whenever cells are deprived from cholesterol. This process has been described to be controlled via activation of the sterol response element binding protein 2 (SREBP2), a transcription factor targeting many cholesterol synthesizing enzymes such as HMG-CoA reductase (HMGCR) [22]. We measured gene expression of well-known SREBP2 targets, responsible for increased cholesterol uptake and synthesis, but we did not observe significant regulation. Instead we found a translation independent, HMGCR protein increase, which correlated with increased cholesterol synthesis. This reflects a novel control of cholesterol synthesis on the protein level and does not necessarily involve mRNA regulation. So far the effect of HIF1A on HMGCR stability has only been described in human fibroblasts [14,15]. To our knowledge an association of SR-B1 with HIF1A and HMGCR has not been recognized in tumor cells. This indicates that possible therapeutic interventions, involving SR-BI and targeting cholesterol homeostasis, have also not been investigated so far.

Another question is how SR-BI upregulates HIF1A signaling? In endothelial cells SR-B1 was found to regulate AKT phosphorylation [23] and in breast cancer cells a dominant negative AKT mutant inhibited SR-B1 dependend AP-1 activation [24]. Akt activation can contribute to increased HIF signaling [25]. Also, the availability of cholesterol has been associated with increased aerobic glycolysis and increased proliferation in colon cancer [26]. This metabolic program is also closely associated with increased HIF activity. We performed an unbiased search for altered processes after SR-B1 knockdown and found S1P signaling highly regulated. S1P is generated from sphingosine, which is converted to S1P via sphingosine kinase, while degradation of S1P can occur via S1P lyase. For membrane receptor binding S1P is first transported outside of the producing cell [27], then S1P binds to S1P receptors, with S1PR1 and S1PR3 being highest expressed in melanoma. While there are many inhibitors of S1PR, fingolimod stands out because on the one hand it is a potent inhibitor of S1PR1 and S1PR3 and on the other hand it is currently used in patients to dampen inflammatory pathways [28]. Mechanistically, we showed evidence for HIF1A activation to be driven by S1P signaling, which depended on the presence of SR-B1. Interestingly, these findings are supported by previous research performed in endothelial cells, which showed that expression of SR-B1 correlates with COX2 upregulation [29]. 50-70% of plasma S1P is bound to HDL and HDL mediates the interaction between SR-B1 and S1PR1 leading to S1PR signaling in rat aortic smooth muscle cells [30]. Recently, also in macrophages, a direct interaction between SR-B1 and S1P receptor was found [31]. Our findings highlight the pivotal role of SR-B1 in S1P-mediated autocrine stimulation and inflammatory signaling in melanoma, processes that can potentiate the malignant phenotype of cells.

Finally, we generated tumor organoids using human embryonic stem cells and melanoma cell spheroids to test for the efficiency of SR-B1 inhibitors in reducing tumor cell viability in combination with statins, as well as Vemurafenib. Statins work by inhibiting HMG-CoA reductase activity, forcing cells to switch to cholesterol uptake, and are therefore widely used as cholesterol-lowering drugs. By reducing endogenous cholesterol synthesis, statins have also demonstrated potential antitumor effects in various cancer studies, including melanoma [32,33]. Similarly, targeting SR-B1 using specific inhibitors like BLT1 can effectively reduce cholesterol uptake, disrupting the metabolic requirements of cells [34]. Both statins and SR-B1 inhibitors offer promising therapeutic avenues by not only depriving melanoma cells of cholesterol, but also modulating associated signaling pathways involved in cell proliferation, survival, and metastasis. Hence, by carefully combining suitable doses of both drugs, melanoma patients could benefit from such treatment regiments. In the present study human patients were not analyzed. Since, so far, SR-B1 is not targeted in melanoma patients. Instead we relied on human organoids grafted with human melanoma, since these models are more accessible and, as shown here, combinatorial approaches can be tested more easily. We also believe that this approach has benefits when compared to animal experiments.

In summary our study highlights SR-B1 as a key regulator of cholesterol metabolism in melanoma and as a promising therapeutic target for mitigating melanoma progression.

## Methods

### Cell culture and cytotoxicity assay

Human primary melanoma cell line MCM1DLN was generated by Swoboda et al [35]. 1205Lu and WM451Lu were commercially acquired from ATCC (Manassas, VA). Melanoma cells were screened for mycoplasma contamination once a week and routinely cultured in MIM medium supplemented with 2 % FCS (GE Healthcare, Little Chalfront, UK) containing: 80 % MCDB153 (Sigma-Aldrich, St. Louis, MO), 20 % Leibovitz`s l15 (Mediatech, Tewksbury, MA), 5 µg/ml Insulin, bovine (Sigma-Aldrich), 0.5 ng/ml Epidermal Growth Factor (EGF) (Sigma-Aldrich), 1.68 mM CaCl_2_ (Sigma-Aldrich), 100 IU/ml Penicillin (Sigma-Aldrich), 100 µg/ml Streptomycin (Sigma-Aldrich) and 2 µg/ml Ciprofloxacin (Sigma-Aldrich). For the assessment of cytototoxicity after inhibitor treatment, the reduction of resazurin to resorufin cells was measured by using the CellTiter-Blue assay (Promega,Mannheim, Germany) according to the manufacturer's instructions.

### siRNA transfection

siRNA transfection was carried out using DharmaFECT (Thermo Fisher Scientific, Waltham, MA) according to the manufacturer`s protocol. Before the day of transfection, melanoma cells were passaged in antibiotic-free 2 % MIM medium and transfected as soon as the cells were 60-70 % confluent. 20 µM siRNA (GE Healthcare, Little Chalfront UK) was diluted in 1x siRNA buffer (Thermo Fisher Scientific) to a final concentration of 5 µM. 5 µM siRNA was diluted 1:20 in serum free MIM. In a second reaction batch, DharmaFECT (Thermo Fisher Scientific) was diluted 1:50 in serum free MIM. Both reactions were incubated for 5 min at RT. Subsequently, the two reactions were mixed and incubated for 20 min at RT. Afterwards, the mixtures were added to the cells in a ratio 1:5 in MIM medium to a final siRNA concentration of 25 nM. Cells were incubated for 24 h at 37°C in 5 % CO_2_. Hereafter, medium was replaced with 2 % FCS MIM. The following siRNAs was used: SCARB1 siRNA, HIF1A siRNA (GE Healthcare). Non-targeting siRNA (GE Healthcare) was used as a control in all experiments.

### Measurement of cellular lipid content

Gaschromatography was used to directly quantify the cellular content of free cholesterol and cholesteryl esters within a single run [36]. Cells were detached using trypsin, and lipids were isolated from cell pellets by standard Folch extraction. An aliquot of the pellet was used for cell protein determination by the Bradford assay. The analyses were carried out on a GC-2010 gas chromatograph (Shimadzu, Kyoto, Japan) equipped with a programmed temperature vaporizer injector and a fused silica capillary column (DB-5; 12 m length; 0.25 mm inner diameter). Tridecanoyl glycerol and cholesteryl myristate (Sigma-Aldrich) were used as standards for free and esterified cholesterol, respectively. The chromatograms were quantified using GC Solutions 2.3 (Shimadzu), and results were normalized to cell protein.

### Cholesterol synthesis measurement

48 hours after siRNA treatment the cells were trace-labeled with 1 μCi/ml ^14^C-acetate (Perkin Elmer, Waltham, MS, USA) for one hour, lipids were extracted from cell monolayers using hexane/isopropanol (3/2). Directly before lipid extraction, ^3^H-oleic acid was added as recovery marker to the cell monolayers to allow for compensation of sample loss during lipid extraction and TLC. Cells were lyzed using 0.1 mol/l NaOH and cell protein was quantified using the Bradford method. Lipid extracts were saponified using aqueous KOH and non-saponified lipids (i.e. cholesterol and lanosterol) were extracted using hexane. Lipid extracts were concentrated and separated by TLC on silica gel sheets (Polygram SIL G; Marcherey-Nagel, Düren, Germany) using CHCl_3_ as solvent. Spots were detected by iodine vapor (Rf values: 0.23 for free cholesterol and 0.35 for lanosterol), excised, and analyzed by liquid scintillation counting. Values were normalized to the recovery marker and cell protein levels.

### mRNA expression analysis

For SCARB1 mRNA expression in tumor samples, data was obtained from the TCGA consortium [37]. For each melanoma cell line used, RNA was isolated from two biological duplicates, pooled and hybridized to GeneChip Human PrimeView arrays (Affymetrix). CEL files were imported to Affymetrix Expression Console Software and robust multi-array average was calculated. The desktop application version of GSEA was used for gene set enrichment analysis. Following settings were used: Expression dataset: log_2_ RMA normalized expression file; Collapse dataset: true; Permutation type: gene_set; metric of ranking genes: ratio of Classes; collapsing mode: Median_of_probes; normalization mode: meandiv as ranking metric. Scatter plot was made with Affymetrix Transcriptome Analysis Console v2.0 Software. Microarray data and description of experimental design were deposited at NCBI GEO number GSE96743.

### Western blotting

For protein isolation, cells were directly lyzed in whole cell extraction buffer RIPA (Cell Signaling, Danvers, MA) containing 1 mM PMSF (Sigma-Aldrich, St. Louis, MO) and 1 % PIC (Sigma-Aldrich). Samples were centrifuged for 20 min at 16,000 rcf at 4°C. Subsequently, supernatant was collected and stored at -80°C. Protein concentration was measured using the BCA Protein Assay Kit (Thermo Fisher) according to manufacturer`s instructions. 20-40 µg protein per sample was denatured at 95°C for 5 min and loaded onto an acrylamide gel (Bio-Rad, Hercules, CA). Subsequently, the separated proteins were electroblotted onto a nitrocellulose membrane. The following primary antibodies were applied for immunodetection: SR-B1, HIF1a, HMGCR, SGPL1, COX2, SPHK1, beta-actin and alpha-tubulin. On the following day, membranes were incubated with appropriate secondary antibodies for detection by an LiCor Odyssey infrared imaging system (LiCor Biosciences).

### S1P measurement

For intracellular S1P measurement melanoma cells were lyzed in 20 mM PIPES, 150 mm NaCl, 1 mM EGTA, 1% (v/v) Triton X-100, 1.5 mM MgCl_2_, 0.1% SDS, 1 mM sodium orthovanadate, protease inhibitor mixture (without EDTA) at pH 7. The S1P-ELISA (Echelon, ImTec Diagnostics, Antwerpen, Belgium) was performed according to the manufacturer's instructions. Absorbance was measured at 450 nm and the concentration of S1P in the samples was determined by delineation from a standard curve.

### Organoid culture

Skin organoids were generated using human embryonic stem cells WA19 (WiCell CVCL_9780) of early passages (p16-19) according to the protocol of Lee et al. [38]. The only modification to the protocol was using 5000 cells per well for the initial seeding.

### Spheroid formation assay

Metastatic tumor lines were GFP labelled as previously described [39], and 1500 cells were used to generate single tumor spheres in non-tissue-culture-treated, round bottom 96-well plate (SPL Life Sciences) by culturing in media containing 80 % vol DMEM with 5 % FCS and 1 % L-glutamine and 20 % methylcellulose 33 mM. After 48 hours tumor spheroids formed and the spheroid medium was replaced with OMM (organoid maturation medium 49,5% vol Advanced DMEM/F12, 49.5 % Neurobasal medium, 1 % Organoid Matrigel Corning, 100μg/mL Normocyn, 0.1 mM B-Mercaptoethanol, 0,5x N2 supplement, 0.5x B27-VitA supplement, 1x Glutamine).

### Spheroid transplantation

Human skin organoids that were 40 days old were placed on top of tumor spheroids in 96 well U-bottom low-attachment plates (SPL Life Sciences). They remained in direct contact for 48 hours in OMM at 37°C, 5 % CO_2_, with no shaking. After this time point, when the tumor spheroid attached to the organoids, they were transferred to shaking (65 rpm) 24 well ultra-low attachment plates (Corning CLS3473-24EA) and were cultivated for 14 days. Media change was performed every second day. The organoids with attached tumor spheroid are henceforth referred as tumor organoids. Treatment was performed by addition of 100 nM BLT-1, 1000 nM Lovastatin and 500 nM Vemurafenib with every medium change.

### Immunohistochemistry

Tumor organoids were fixed in 4 % paraformaldehyde, washed with 1x PBS and embedded into 1 % agarose molds, processed into paraffin blocks and sectioned. Tissue slides, containing paraffin-embedded samples, were melted for 20 min at 60°C and rehydrated by subsequent incubation in Roticlear, Isopropanol, 96 % Ethanol, 70 % Ethanol and 50 % Ethanol. Then, slides were washed and heated up to 120°C in a pH 6.0 buffer or a pH 9.0 buffer (Dako, Glostrup, Denmark), depending on the antibody. After cooling to room temperature, samples were incubated with 1 % H_2_O_2_ (Sigma-Aldrich, St. Louis, MO) for 15 min. Afterwards, samples were permeabilized with 0.1 % TritonX-100 (Sigma-Aldrich) for 5 min. Then, sections were blocked with 1 % BSA/PBS for 1 h (Vector Laboratories, Burlingame, CA) at room temperature. Subsequently, sections were incubated over night at 4°C with the primary antibodies directed against GFP, Ki67, cleaved caspase 9. On the next day, slides were washed and the secondary anti-rabbit antibody (Vector Laboratories) was added for 45 min at room temperature. After a washing step, sections were incubated for 30 min with Streptavidin-HRP (Leica, Wetzlar, Germany). For detection, slides were incubated with AEC+ High Sensitivity Substrate Chromogen (Dako). Counterstaining with hematoxylin solution was performed according to Mayer (Carl Roth, Karlsruhe, Germany); arrays were mounted with Aquatex® (Merck Millipore, Billerica, MA).

### Statistics

For bar diagrams, standard error of the mean (SEM) and two tailed P-values were calculated by performing unpaired (independent) Student`s or Welch`s t-test in SPSS v21 (IBM, Armonk, NY). Levene`s test with a threshold of 0.05 was performed to choose the appropriate t-test. For multiple comparisons, One-way ANOVA was calculated. Tukey HSD was used for post-hoc analysis. For line diagrams, standard deviation (SD) was calculated in GraphPad Prism 6 (GraphPad Prism Inc., La Jolla, CA). Two tailed P-values were calculated by performing unpaired (independent) Student`s or Welch`s t-test in SPSS to compare individual time points and the areas under the curve (AUC). Levene`s test with a threshold of 0.05 was performed to choose the appropriate t-test. For multiple comparisons, One-way ANOVA was calculated.

For scatter plots Pearson correlation analysis was applied to calculate corresponding R- and p-values in SPSS.

## Funding

**This work was supported by the Austrian Science Fund, FWF, grant number P32979 and P35387-B (to Mario Mikula).**

## Data availability

The transcriptomic data used in this publication have been deposited in NCBI's Gene Expression Omnibus (GEO Series accession numbers GSE96743). Other data and materials in this paper are available upon reasonable request.

## CRediT authorship contribution statement

**Oliver Eckel:** Writing – review & editing, Visualization, Methodology, Investigation, Formal analysis. **Madalina A. Mirea:** Validation, Methodology, Investigation. **Anna Gschwendtner:** Visualization, Methodology, Investigation. **Martina Pistek:** Visualization, Methodology. **Katharina Kinslechner:** Visualization, Methodology. **Clemens Röhrl:** Methodology, Investigation. **Herbert Stangl:** Writing – review & editing, Supervision. **Markus Hengstschläger:** Writing – review & editing, Supervision. **Mario Mikula:** Writing – original draft, Supervision, Project administration, Funding acquisition, Conceptualization.

## Declaration of competing interest

The authors declare that they have no known competing financial interest or personal relationships that could have appeared to influence the work reported in this manuscript.

## Appendix Supplementary materials

Image, application 1
