# Supplementary material for: Expression of the cholesterol transporter SR-B1 in melanoma cells facilitates inflammatory signaling leading to reduced cholesterol synthesis
Source: Neoplasia. 2025 Mar 21;63:101154. doi: 10.1016/j.neo.2025.101154 (PMC11981749; doi:10.1016/j.neo.2025.101154)
Supplement: Supplementary file 1 [file mmc1.pdf]

Supplementary Figure 1

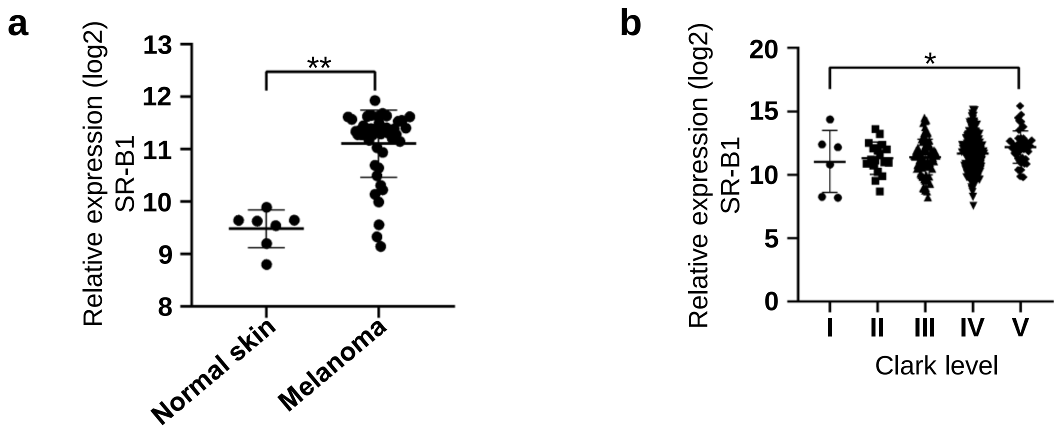

A) SR-B1 mRNA expression in normal skin tissue, primary melanoma and melanoma metastatic samples. B) Comparison of SR-B1 mRNA levels in malignant melanoma tissue diagnosed with indicated clark levels. All data from the GSE3189 dataset. * = P < 0.05, ** = P < 0.01.

Supplementary Figure 2

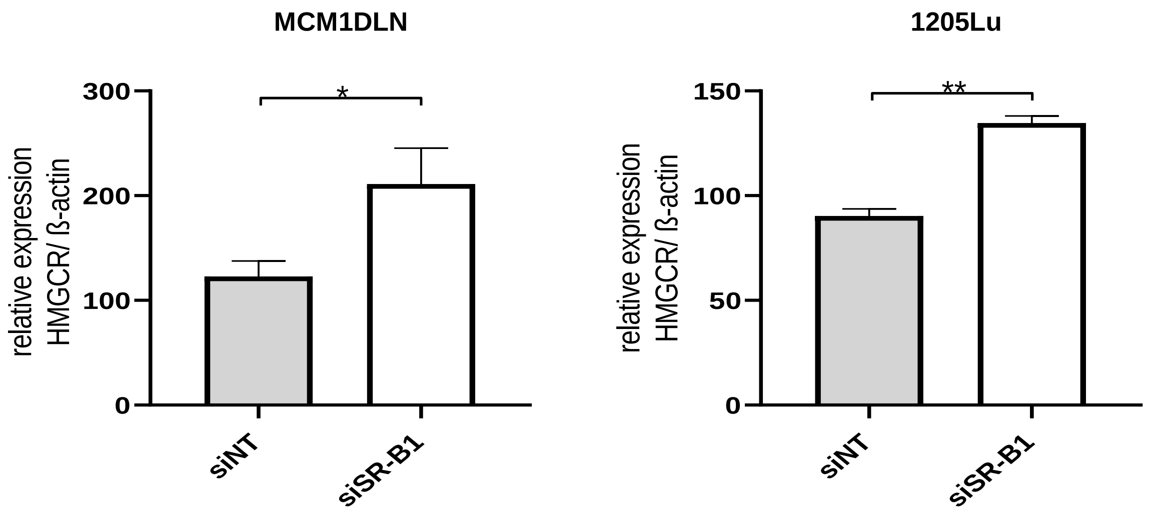

Quantification of normalized protein amounts of HMGCR measured in triplicate 48 hours subsequent to control or siRNA SR-B1 treatment by western blot. * = P < 0.05, ** = P < 0.01.

Supplementary Figure 3

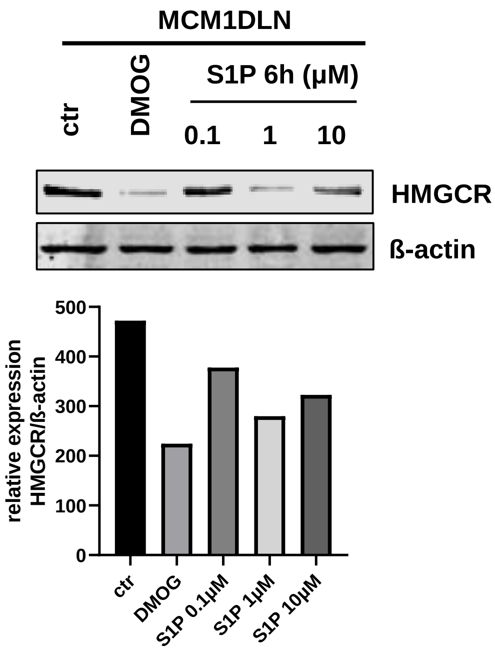

Western blotting was performed to measure the amounts of HMGCR after exposure to S1P with the indicated concentrations. DMOG was used as a positive control. Normalized protein quantification displayed in barchart..

Supplementary Figure 4

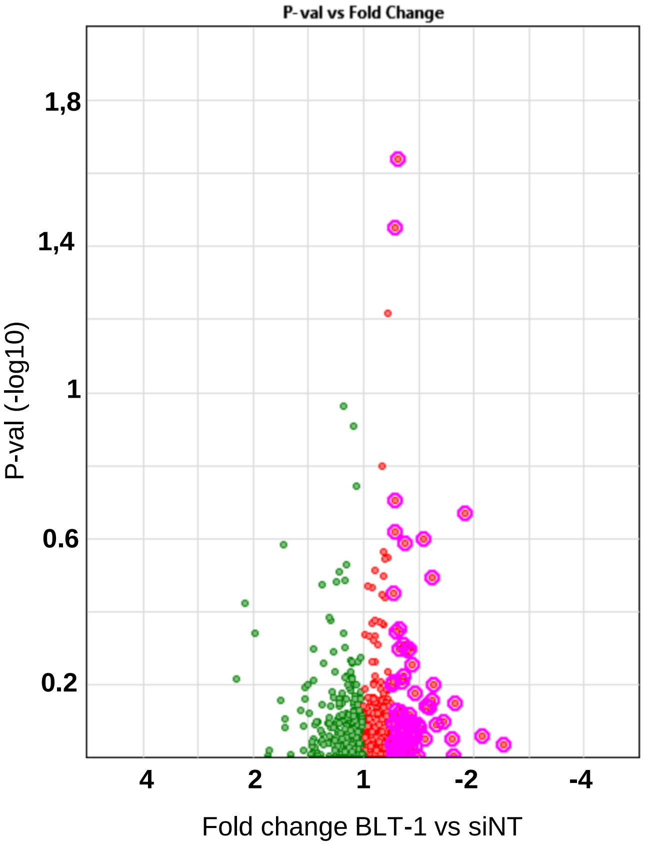

MCM1 DLN and 1205Lu cells were treated with 100 nM BLT-1 for 48 hours. Fold change regulation of genes in the geneset TNFA signaling via NFkB is displayed.

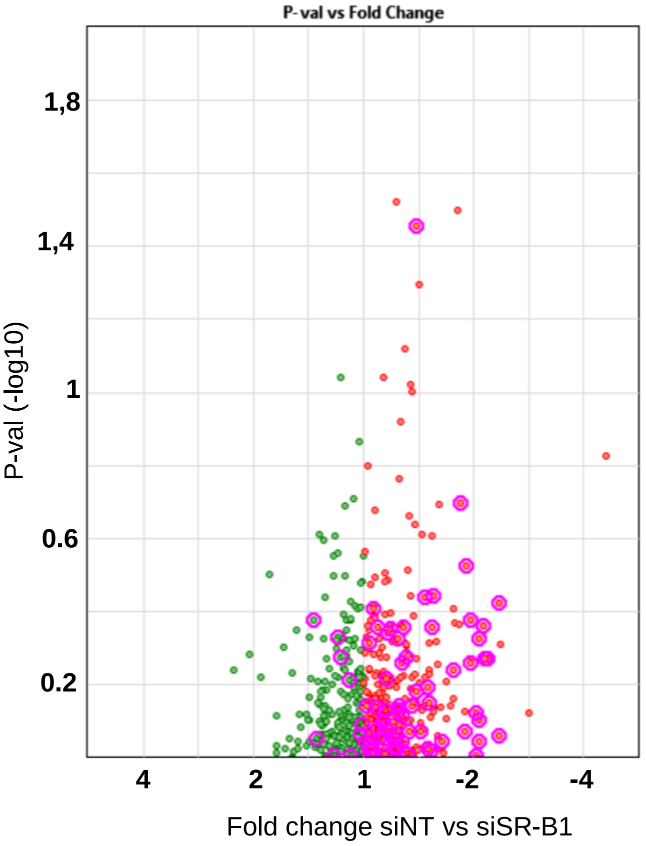

MCM1 DLN and 1205Lu cells were treated with si SR-B1 for 48 hours. Fold change regulation of genes in the geneset TNFA signaling via NFkB is displayed.

KLF2	IER5	EGR2	FOS
IFIH1	SDC4	DENND5A	EGR1
TNFAIP6	JUN	EGR3	TNFAIP2
GLUT14	PTPRE	GFPT2	TIPARP
LIF	INHBA	JAG1	PER1
PHLDA2	KLF2	BTG2	EGR3
EGR1	MCL1	EFNA1	BCL3
MCL1	SOD2	ICAM1	PLPP3
NFKB2	PMEPA1	TANK	CFLAR
ID2	BMP2	PLK2	HES1
CEBPD	HBEGF	PTPRE	ACKR3
ICAM1	GADD45B	OLR1	CSF1
IL7R	**SPHK1**	PFKFB3	ICOSLG
CDKN1A	EGR1	PDE4B	TNFAIP2
CFLAR	DUSP5	MAFF	SLC2A6
EGR2	CDKN1A	ACKR3	MAP3K8
RCAN1	**PTGS2**	PLAU	SMAD3
GCH1	NR4A2	TNFRSF9	CFLAR
CCL2	OLR1	HES1	GPR183
CEBPD	PDLIM5	PER1	CFLAR

List of NFkB hallmark genes, depicted in pink in the above volcano blot, downregulated after BLT-1 treatment as well as after si SR-B1 treatment.

Supplementary Figure 5

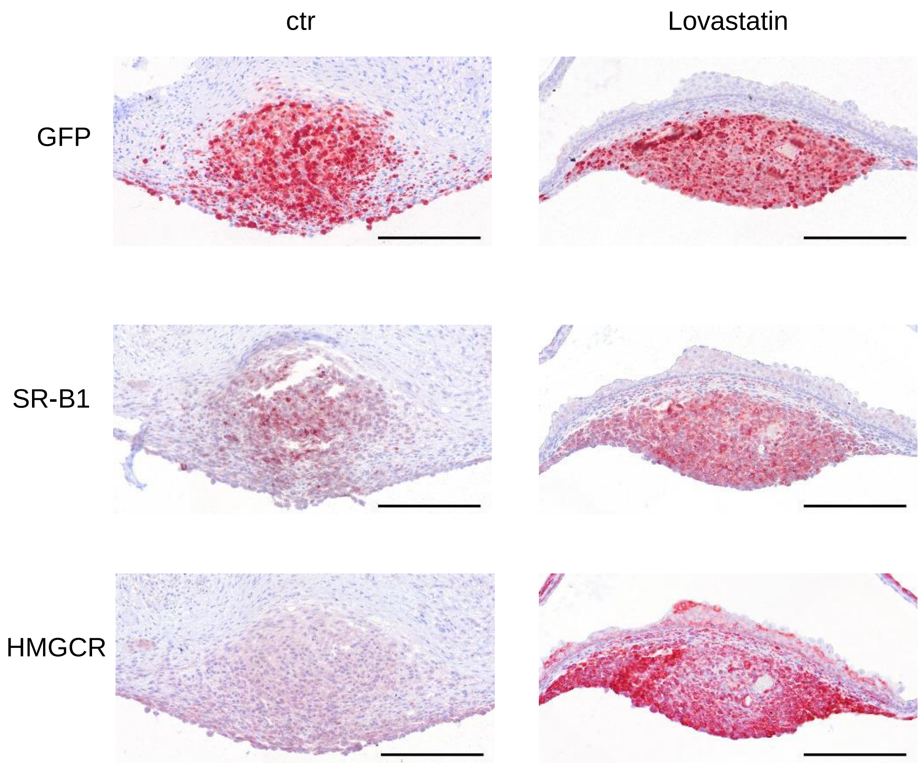

Organoids with transplanted 1205Lu tumorspheres were grown for 14 days without (ctr) or with 1000nM of Lovastatin. Tissue was fixed in 4% formaldehyde, paraffin embedded, sectioned and stained with respective antidodies. Antibody binding was visualized by AEC development (red color), nuclei are hematoxylin stained (blue color). Bar = 100 µm.
